# Laboratory practice on detection of antiphospholipid antibodies: UK NEQAS blood coagulation survey—2024

**DOI:** 10.1111/bjh.70112

**Published:** 2025-08-26

**Authors:** Deepa J. Arachchillage, Mike Laffan, Will Lester, Steve Kitchen, Ian Jennings

**Affiliations:** ^1^ Department of Haematology Imperial College Healthcare NHS Trust London UK; ^2^ Centre for Haematology, Department of Immunology and Inflammation Imperial College London London UK; ^3^ Department of Haematology University Hospitals Birmingham Birmingham UK; ^4^ UK NEQAS for Blood Coagulation Sheffield UK

**Keywords:** activated partial thromboplastin time, antiphospholipid antibodies, antiphospholipid syndrome, dilute Russell's viper venom time, lupus anticoagulant, National External Quality Assessment Scheme

To the Editor,

Clinical diagnosis of antiphospholipid syndrome (APS) is based on updated Sapporo criteria^1^ which were initially designed for the inclusion of patients in clinical studies. The presence of thrombosis (venous, arterial or microvascular) and/or pregnancy morbidities  in association with persistently positive (≥two occasions 12 weeks apart) antiphospholipid antibodies (aPL) fulfils the diagnosis of APS.^1^ The aPL in the routine laboratory diagnostic criteria comprise lupus anticoagulant (LA), IgG or IgM anticardiolipin (aCL) and anti‐β2‐glycoprotein I (anti‐β2GPI) antibodies.

Recently published American College of Rheumatology (ACR)/European Alliance of Associations for Rheumatology (EULAR) classification criteria for APS^2^ aim to maximise specificity for inclusion of APS in clinical studies. They also specify that the solid‐phase aPL should be detected by enzyme‐linked immunosorbent assay (ELISA). As thrombosis and pregnancy morbiditiesare common and contributed by diverse causes, the accurate diagnosis of APS is heavily and critically dependent on laboratory detection of aPL.

Several guidelines provide specifications on aPL testing^3^, ^4^ but despite numerous efforts, standardisation of solid‐phase aPL detection has not been successful. We have previously assessed the compliance and the diagnostic accuracy of centres following recommendations for LA testing in previous guidelines.^5^ The British Society for Haematology (BSH) updated the guidelines on the diagnosis and management of APS in September 2024 and included recommendations for laboratories performing aPL testing.^3^

In this multicentre international survey of members of the UK National External Quality Assessment Scheme (NEQAS) of Blood Coagulation, we aimed to assess the practice of aPL testing using a questionnaire (Supporting Information, pp. 1–3), formulated by the authors by consensus prior to the publication of the 2024 BSH guidelines. The questionnaire was sent on two occasions in January 2024 and July 2024 respectively.

Of the 252 centres participating in NEQAS of blood coagulation, 81 (32.1%) responded to the survey. Of these, 60 responses were received in January, and a further 21 were received in July 2024. Not all 81 centres answered every question. Twenty‐nine of the 81 centres were located outside the United Kingdom.

Table S1 summarises the information on sample preparation for LA testing. Double centrifugation to obtain platelet‐poor plasma with a platelet count <10 × 10^9^/L is required prior to LA testing^3^ and local verification is suggested to ensure this requirement is met in a sample size of ≥20 following double centrifugation.^3^ Surprisingly, of the 81 centres, only 62 (76.5%) double centrifuged the samples and then nine checked the platelet count on all samples prior to LA testing. Of concern, nine centres perform LA without double centrifugation, which can lead to false‐negative LA results due to the presence phospholipid from platelets. Around 50% of centres accepted samples from other centres for LA testing, and there was great variation in the time limit on acceptance of those samples (2–24 hrs). Samples for LA should be double centrifuged, ideally within 4 hrs of sample collection,^6^ except samples for dilute Russell's viper venom time (DRVVT) are stable at room temperature for up to 24 hrs before double centrifugation and freezing.^7^ However, activated partial thromboplastin time (APTT) should be performed within 4 hrs of sample collection.^8^ If transport times exceed 4 hrs, laboratories should verify sample stability and adapt their testing repertoire and/or reporting comments where necessary. It is recommended to reject haemolysis/icterus/lipaemia (HIL) samples for aPL tests, as this can usually cause false negatives^9^ unless catastrophic APS with intravascular haemolysis is suspected,^6^ where results should be reported with caution; four centres accepted HIL samples for aPL testing.

Forty‐three centres (53%) accepted samples from other centres for LA testing, while 35 (43.2%) centres performed LA only from local requesters. Of the 43 centres accepting samples from other centres, 31 (72.1%) had a time limit for the acceptance of samples since sample collection. There was wide variation in this time limit (2–24 hrs) (Table S1). A majority of centres (50/81, 61.7%) rejected samples if there was HIL while a minority (4/81, 4.9%) accepted them. Of the remaining 27 centres, 13 centres did not respond to the question, while the rest accepted icteric/lipaemic samples but not haemolysed samples.

There was variable practice in the use of LA‐sensitive and LA‐insensitive reagents for screening (Table 1). Most centres (77/81, 95.1%) performed DRVVT in all samples. A small number of centres performed Silica clotting time, Kaolin clotting time and diluted PT (Table 1). Details of the APTT testing for the presence of LA are summarised in Table S2. Only 36/41 responding centres used two APTT reagents in all samples, while five centres used two APTT reagents only on selected samples. If the laboratory used two APTT reagents, an algorithm was used to compare the results by only 12 centres. Three centres (5%) performed APTT mixing studies irrespective of APTT prolongation while 56 (95%) performed mixing only if APTT was prolonged. An algorithm was used to determine the correction of mixing studies in 25/59 centres.

**TABLE 1:** Tests employed when screening for lupus anticoagulant.

Test	Number^a^
Prothrombin time	All patients *n* = 55, selected patients *n* = 4
Thrombin time	All patients *n* = 15, selected patients *n* = 9
Fibrinogen level	All patients *n* = 32, selected patients *n* = 3
Two APTT reagents on all patients	36
Two APTT reagents on selected patients	5
One LA‐sensitive APTT reagents on all patients	34
One LA‐insensitive APTT reagent on all patients	9
One LA‐insensitive APTT reagent on selected patients	2
DRVVT on all patients	77 (4 did not answer)
SCT on all patients	21 in all samples and 3 in selected samples
KCT	2
Dilute PT	2

Abbreviations: APTT, activated partial thromboplastin time; DRVVT, diluted Russell's viper venom test; KCT, Kaolin clotting time; LA, lupus anticoagulant; SCT, Silica clotting time.

^a^ Total number is 81 centres and the number indicated in the table next to each test is the number of centres performing each test. Not all centres answered every question.

A large majority, 78 centres (96.3%), performed DRVVT on all LA requests. Of the 78, 18 did both screen and confirm tests with high phospholipid concentration on all samples for LA testing (23.1%) while others performed confirm testing only if the DRVVT screen test was prolonged. Several different algorithms were used to interpret the DRVVT screen and confirm results, sometimes in combination (Table 2). Of 81 responses, 42 centres (51.9%) did not do mixing studies for their DRVVT, while 39 centres (48.1%) performed mixing studies on at least some samples (8/39 in all samples and 31/39 in some samples). It is recommended to generate in‐house cut‐off values for positive results for DRVVT using the 99th centile or verification of a manufacturer's cut‐off (using the same reagent, analyser and pooled normal plasma as used by the manufacturer). In fact, 41 centres used the manufacturer's cut‐off and others used literature values (9) but only 20 used in‐house data, sometimes in combination.

**TABLE 2:** Diluted Russell's viper venom testing.

Testing scenario	Yes	No
Perform DRVVT on all lupus anticoagulant samples	78	3
Perform screen test and confirm (high phospholipid) DRVVTs on all lupus anticoagulant samples	18	53 (only performed if DRVVT screen prolonged)
Algorithms to use interpret DRVVT results		
1. Test/normal ratio	37	
2. Confirm/normal ratio	32	
3. Test/confirm ratio	27	
4. Normalised test/confirm ratio	41	
5. % correction	1	
6. % correction ratio	11	
7. Combination of above	39	
Ten different combinations of algorithms are employed, the most common being test/normal ratio, confirm/normal ratio and test/confirm ratio		
Perform mixing studies as part of your DRVVT screen	All DRVVTs *n* = 8 Some DRVVTs *n* = 31	42
Definition of a positive or abnormal DRVVT result come from		
1. Manufacturer	41	
2. Literature	9	
3. In house	20	
4. Other including combinations of above	11	

Abbreviation: DRVVT, diluted Russell's viper venom test.

The majority of centres (78/81) used both positive and negative internal quality controls (IQC) while two centres used only negative IQC, and the remaining centre only used a positive IQC.

Although the guidelines strongly recommend that laboratories should report detailed quantitative results for all aPL tested alongside an interpretation of results, this was followed only by 45 centres when reporting LA while negative/borderline/positive (or equivalent wording) was used by 24 centres, and 11 centres provided only numerical values without interpretation of the results. If LA testing result interpretation was reported as equivocal, 28 centres responded that they request a repeat in 12 weeks to verify the results.

Although guidelines recommend that coagulation screen including PT, APTT and Clauss fibrinogen should be performed as a screen on all LA requests, this was not followed by all centres (Table 1).

LA testing while on anticoagulation often causes false‐positive results and rarely can cause false‐negative results in patients on apixaban^10^ leading to overdiagnosis or underdiagnosis of APS with potential risk of serious harm to the patients from bleeding or recurrent thrombosis. While LA testing on anticoagulation is strongly discouraged and testing for LA should ideally be postponed until anticoagulation has been discontinued for a suitable time period, if it is essential to perform LA, several approaches are suggested by the current BSH guidelines^3^ and others.^11^ Samples should be collected at a treatment trough and drug levels measured to confirm that levels are too low to interfere with LA testing. Commercial reagents containing activated charcoal, and collectively called ‘direct oral anticoagulants (DOACs) neutralisers’, have been developed to remove the effect of DOAC.^11^ Use of these activated charcoal products (ACP) for LA assays in samples from individuals on direct factor Xa inhibitors and direct thrombin inhibitors showed promising results.^12^ However, they are not effective against heparinoids or vitamin K antagonists. It is therefore of concern that 26 centres (32%) tested samples from anticoagulated patients without pretreatment whereas 43% did not test these samples. Four centres (5%) used heparinase to neutralise any heparin effect while 22 centres (27%) used DOAC stop/remove tests to eliminate the effect of DOACs.

Historically, all solid‐phase aPL were detected using ELISA, with heterogeneity in analytical platforms and reagents resulting in method variability.^13^, ^14^ The increasing use of automated platforms allows consistent application of protocols, reduces manpower demand and inter‐laboratory variation. However, calibration has been a major issue due to the lack of uniformity of reference material.^15^ Only 42 centres stated that they performed solid‐phase assays of which 69.0% used ELISA and 21.4% chemiluminescence (Figure S1). Despite the recommendation for establishing a local cut‐off value for positivity, many centres (36/42) used the manufacturer's cut‐off for the detection of anti‐β2GPI and aCL antibodies.^3^, ^4^

In conclusion, based on the survey data, it is clear that there was a large variation in the laboratory practice of aPL testing among NEQAS laboratories prior to recently published BSH guidelines on the diagnosis and the management of APS.^3^ However, the overall response rate to the survey was only 32.1%. Some variations such as performing LA testing in patients on anticoagulation and failure to double centrifuge the samples prior to LA testing have significant clinical consequences. The time range for accepting samples for LA testing is also a concern. A repeat survey will be carried out in due course to assess the impact of the updated BSH guidelines with detailed guidance on detection of aPL.

## AUTHOR CONTRIBUTIONS

DJA initiated and designed the survey, interpreted the data and wrote the manuscript. ML interpreted the data and reviewed the manuscript. WL, SK and IJ initiated and designed the survey, interpreted the data and revised the manuscript.

## CONFLICT OF INTEREST STATEMENT

DJA received research reagents from Werfen outside this research and funding to attend national meetings from Werfen and Sysmex. Others have no potential conflict of interest to declare.

## Supporting information


Data S1.
