# Supplementary material for: Laboratory practice on detection of antiphospholipid antibodies: UK NEQAS blood coagulation survey—2024
Source: Br J Haematol. 2025 Aug 26;207(5):2200–3. doi: 10.1111/bjh.70112 (PMC12624191; doi:10.1111/bjh.70112)

**Supplementary materials**

**SECTION 1:  Sample Preparation**

**What sample volume do you normally request/receive for a lupus anticoagulant/aPL investigation?**

**Do you double centrifuge all samples before testing?**

**What speed and time do you centrifuge for?**

**Do you check the platelet count on all samples before testing?**

**Do you accept referral samples from other centres?**

**If you accept referral samples, do you put a time limit on acceptance?  If yes, please state time and whether this applies to all or selected tests?**

**Do you accept samples with raised HIL (haemolysis/icterus/lipaemia) flags?**

**SECTION 2:  aPL TEST REPERTOIRE**

**To investigate a patient for aPL in your centre, which of the following do you perform? (please tick all that apply, leave blank if not performed**

2 APTT reagents on all patients

2 APTT reagents on selected patients

1 LA sensitive APTT reagents on all patients

1 LA insensitive APTT reagents on all patients

1 LA insensitive APTT reagents on selected patients

DRVVT on all patients

SCT on all patients, selected patients

KCT

Dilute PT

PT all patients, selected patients

TT all patients, selected patients

Fgn all patients, selected patients

**Are solid phase assays performed in your department or institution?**

Performed in my lab

Performed in a different department

Solid phase assays not performed in my institution

**Please indicate below which solid phase assays are performed (please tick all that apply)**

ACLA IgG performed on all patients, selected patients

ACLA IgM performed on all patients, selected patients

B2GP1 IgG performed on all patients, selected patients

B2GP1 IgM performed on all patients, selected patients

**SECTION 3:  APTT**

**If you use 2 APTT reagents, do you use both for all aPL investigations?**

**If you use 2 reagents, do you use an algorithm to compare the results?  Please tick any relevant box below.**

No - we just report the results for each reagent

Yes - we report the difference in secs (delta) between the two results

Yes - we report the % difference between results

Yes - we determine a Rosner Index

Yes - other algorithm

**Do you perform mixing studies on your APTTs for LA investigations?**

Yes performed with every APTT

Yes, only if APTT is prolonged

No

**If you perform mixing studies, do you use an algorithm to determine whether the APTT is corrected?**

**SECTION 4:  DRVVT**

**Do you perform a DRVVT on all aPL investigations?**

**Do you perform test and confirm (high phospholipid) DRVVTs on all samples:**

**Which of these algorithms do you use to interpret DRVVT results? (please tick all that apply)**

1. Test/normal ratio

2. Confirm/normal ratio

3. Test/confirm ratio

4. Normalised test/confirm ratio

5. % correction

6. % correction ratio

**Do you perform mixing studies as part of your DRVVT screen?**

Yes on all investigations

Yes on some

No

**Where does your definition of a positive or abnormal DRVVT result come from?**

Manufacturer

Literature

In house

Other

**SECTION 5:  Solid Phase Assays**

**If you perform solid phase assays, which test principle do you use for your assays?**

Enzyme-linked immunoassay

Chemiluminescence

Multiplex immunoassay

Other

**Please indicate the source of your cut-off value for a positive solid phase assay result**

Manufacturer

Locally established

**SECTION 6: Other Questions**

**Do you pre-treat samples for known anticoagulants?**

We use heparinise

We use protamine

We use DOAC stop/remove

We test samples if the patient is on known anticoagulation but dont pre-treat

We dont test any samples if the patient is on known anticoagulation

**Do you use positive and negative controls for internal quality control (IQC)**

We use both positive and negative controls for IQC

We only use negative controls for IQC

We only use positive controls for IQC

**How do you report results?**

Negative / Positive (or equivalent wording)

Negative / Borderline / Positive (or equivalent wording)

Other

**If you report an interpretation as equivocal, how do you define this?**

**Table-S1. Sample preparation for lupus anticoagulant screen and confirmation**

| **Specification** | **Yes** | **No** | **No response or deviation from straightforward yes or no** |
| --- | --- | --- | --- |
| Double centrifuge of the sample prior to testing | 62 | 9 | 10 no response |
| Check the platelet count on all samples prior to testing | 9 | 72 | 0 |
| Accept referral samples from other centres | 43 | 35 | 3 no response |
| If accept referral samples, time limit on acceptance | 31  Time limits:  2hrs n=2  4hrs n=18  6hrs n=2  8hrs n=1  12hrs n=1  24hrs n=3  4 not stated | 12 | Not applicable |
| Do you accept samples with raised HIL | 4 | 50 | 13 no response  14 accept icteric/lipaemic samples |

HIL=haemolysis/icterus/lipaemia

**Table-S2**. **APTT for the lupus anticoagulant test**

| **Testing scenario** | **Yes** | **No** |
| --- | --- | --- |
| 2 APTT reagents, used when testing for LA in all samples | **36** | **5 (only selected samples)** |
| When using 2 APTT reagents, an algorithm is used to compare the results | 12  difference in secs (delta) between the two results n=1  % difference between results n=2  Rosner Index n=3  other algorithm n=6 (including ratio of the two reagent results) | **29** |
| Mixing studies are performed on APTT for LA testing | **59**  With every APTT n=3  Only if APTT prolonged n=56 | **22** |
| Use an algorithm to determine whether the APTT is corrected if mixing studies are performed | **25**  **(**Including ICA%, Rosner index, correction to within or close to normal range**,** | **34** |

APTT= activated partial thromboplastin time; LA= lupus anticoagulant; ICA= index of circulating anticoagulant


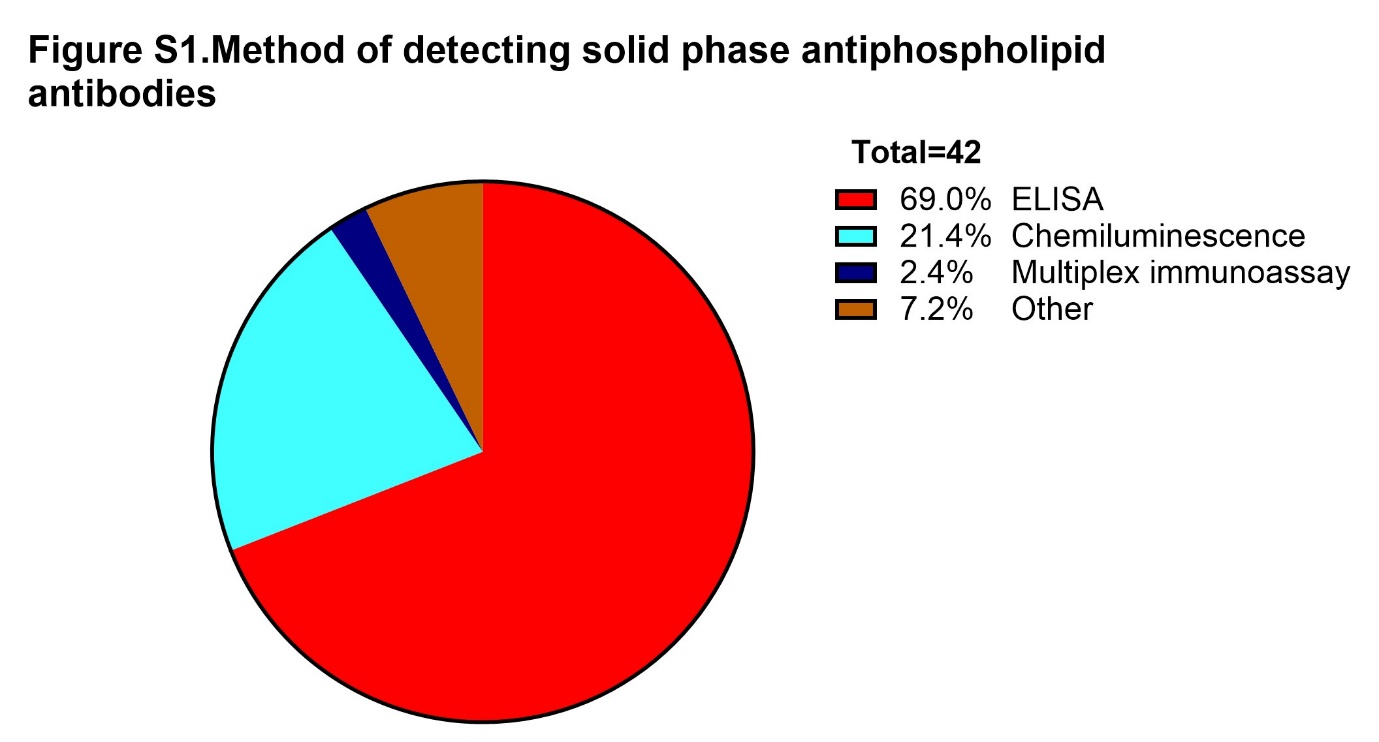

Supplement: Supplementary file 1 — Data S1. [file BJH-207-2200-s001.docx]
